# Translational independence between overlapping genes for a restriction endonuclease and its transcriptional regulator

**DOI:** 10.1186/1471-2199-11-87

**Published:** 2010-11-19

**Authors:** Meenakshi K Kaw, Robert M Blumenthal

**Affiliations:** 1 Department of Medical Microbiology and Immunology, University of Toledo Health Science Campus, 3100 Transverse Drive, Toledo, OH 43614-2598, USA; 2 Program in Bioinformatics and Proteomics/Genomics, University of Toledo Health Science Campus, 3100 Transverse Drive, Toledo, OH 43614-2598, USA

## Abstract

**Background:**

Most type II restriction-modification (RM) systems have two independent enzymes that act on the same DNA sequence: a modification methyltransferase that protects target sites, and a restriction endonuclease that cleaves unmethylated target sites. When RM genes enter a new cell, methylation must occur before restriction activity appears, or the host's chromosome is digested. Transcriptional mechanisms that delay endonuclease expression have been identified in some RM systems. A substantial subset of those systems is controlled by a family of small transcription activators called C proteins. In the PvuII system, C.PvuII activates transcription of its own gene, along with that of the downstream endonuclease gene. This regulation results in very low R.PvuII mRNA levels early after gene entry, followed by rapid increase due to positive feedback. However, given the lethal consequences of premature REase accumulation, transcriptional control alone might be insufficient. In C-controlled RM systems, there is a ± 20 nt overlap between the C termination codon and the R (endonuclease) initiation codon, suggesting possible translational coupling, and in many cases predicted RNA hairpins could occlude the ribosome binding site for the endonuclease gene.

**Results:**

Expression levels of *lacZ *translational fusions to *pvuIIR *or *pvuIIC *were determined, with the native *pvuII *promoter having been replaced by one not controlled by C.PvuII. In-frame *pvuIIC *insertions did not substantially decrease either *pvuIIC-lacZ *or *pvuIIR-lacZ *expression (with or without C.PvuII provided *in trans*). In contrast, a frameshift mutation in *pvuIIC *decreased expression markedly in both fusions, but mRNA measurements indicated that this decrease could be explained by transcriptional polarity. Expression of *pvuIIR-lacZ *was unaffected when the *pvuIIC *stop codon was moved 21 nt downstream from its WT location, or 25 or 40 bp upstream of the *pvuIIR *initiation codon. Disrupting the putative hairpins had no significant effects.

**Conclusions:**

The initiation of translation of *pvuIIR *appears to be independent of that for *pvuIIC*. Direct tests failed to detect regulatory rules for either gene overlap or the putative hairpins. Thus, at least during balanced growth, transcriptional control appears to be sufficiently robust for proper regulation of this RM system.

## Background

Bacterial type II restriction-modification (RM) systems are abundant in both the bacterial and the archaeal worlds [1]. Many play important roles in defense against phage [2], but they also appear to modulate horizontal gene transfer [3], and to act as "selfish" toxin-antitoxin addiction modules [4,5]. Type II RM systems generally specify separate DNA methyltransferase (MTase) and restriction endonuclease (REase) proteins [6]. Many type II RM systems are on mobile genetic elements, but even chromosomal RM systems can move into new host cells via transduction, transformation or conjugation [7-10].

PvuII is a plasmid-borne type II RM system from the Gram-negative bacterium *Proteus vulgaris *[11]. The MTase (M.PvuII) modifies the cognate DNA sequence CAGCTG by methylating the internal cytosine [12], generating N4-methylcytosine [13]; while the REase (R.PvuII) cleaves the central GpC if this sequence is unmethylated [13-15]. The REase and MTase act independently of each other in type II RM systems. As a result, strict regulation is needed after the genes enter a new cell in order to delay REase accumulation until the MTase has had time to protect the new host's DNA. The basis for this regulation is unknown for most RM systems.

A subset of type II RM systems are controlled by a third gene, that was designated as "C" (controller) protein when first discovered in the BamHI and PvuII systems [16,17]. Sequence comparisons quickly identified orthologs in the SmaI and EcoRV systems [17], and since then C proteins have been identified (and in some cases confirmed) in a wide variety of other RM systems [18].

The transcriptional regulation of C-controlled RM systems is understood in outline, from the structure of the C proteins [19-22], through their action at conserved "C boxes" upstream of the C genes [7,9,23-27], to their dual function as activators and repressors [24] and possible interaction with RpoD (σ^70^) [7,20]. The temporal behavior of one of these systems has also been studied, following its introduction into new host cells [28].

**Figure 1:**
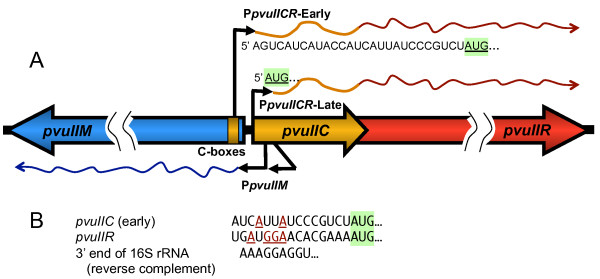
**PvuII transcripts**. **A**. The diagram indicates the expected transcripts early or later after the PvuII genes enter a new host cell. The timing is inferred from *in vivo *transcript mapping in the presence or absence of active C.PvuII [7], and following synchronous infection of cells with bacteriophage carrying the PvuII genes [28]. "C boxes" are the binding sites for the autogenously-activating C protein. The two *pvuIIM *(methyltransferase) promoters appear to be constitutive. **B**. The ribosome-binding (Shine-Dalgarno) sequences are predicted based on location relative to the *pvuIIC *start codon (shaded green), and comparison to the Logo for ribosome binding sites adapted from [32].

The PvuII genes naturally reside on a mobilizable plasmid [8,12,29]. When these genes enter a new host cell with no pre-existing C.PvuII protein, the MTase gene (*pvuIIM*) is rapidly transcribed from a pair of C-independent promoters [7,28] (Figure 1A). The REase gene (*pvuIIR*) has no separate promoter, and depends on two promoters upstream of the C gene *pvuIIC *[7]. One of these two promoters is weak but independent of C.PvuII. The resulting transcript begins 26 nt upstream of the *pvuIIC *initiation codon [7] (Figure 1A), so translation relies on a ribosome binding site (RBS, also called a Shine-Dalgarno sequence) [30,31]. Because most of our experiments are carried out in *E. coli*, it is important that the 3' 60 nt of 16 S rRNA, which includes the "anti-Shine-Dalgarno" sequence complementary to the RBS, is identical in *E. coli *and *P. vulgaris *(*e.g*., compare GenBank accession numbers S000629954 with X07652 or J01874).

The RBS for *pvuIIC *is very poor (Figure 1B), based on the very limited similarity to an AGGAGG motif [32]. The expected poor translation initiation would synergize with the lack of transcriptional autogenous activation by C.PvuII [17], by slowing the elongation of any transcripts that did get produced [33]. In contrast, when C.PvuII eventually begins to accumulate, it activates transcription from the second promoter, rapidly boosting transcription of *pvuIIC *and *pvuIIR *via a positive feedback loop [7,28]. Furthermore, the resulting C.PvuII-dependent transcript is leaderless [7], beginning at the *pvuIIC *initiator codon, and is thus independent of the RBS [34-36].

A key area of uncertainty in this model is whether transcription-level regulation is sufficient to protect the cell. Early transcription from the weak C-independent promoter appears to proceed into *pvuIIR *[28]. If *pvuIIR *mRNA can be translated well, independent of the rate at which the upstream *pvuIIC *gene is translated, this might lead to unacceptably rapid early accumulation of restriction activity. Unlike *pvuIIC*, *pvuIIR *has an obvious RBS motif (Figure 1B). We accordingly investigated whether there is some form of translational control of *pvuIIR*.

**Figure 2:**
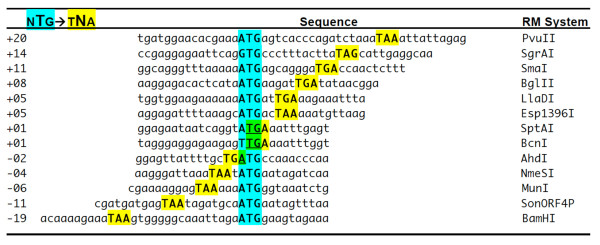
**Relative Locations of C Terminators and R Initiators of Translation**. Selected C.PvuII orthologs that are upstream of known or candidate restriction endonuclease genes were aligned via the endonuclease initiation codons to illustrate the range of relative positions. Names and gene boundaries are available at REBase [1]. Numbers at the left are center-to-center distances between the C gene termination codon and the R gene initiation codon.

We were particularly intrigued by two features of the PvuII genes: first, the *pvuIIC *and *pvuIIR *genes overlap, and second, *pvuIIR *is preceded by potential hairpins, one of which would occlude the *pvuIIR *RBS. The *pvuIIC *and *pvuIIR *genes are cotranscribed [9], and many polycistronic transcripts require translation of an upstream gene for efficient translation of a subsequent gene. This phenomenon, resulting from poor loading of free ribosomes onto the internal translation initiator, is called translational coupling, and was first observed in the tryptophan operon of *E. coli *[37]. Coupling might play an important role in delaying REase accumulation in C-controlled RM systems, relative to that of MTase, after the genes for the system enter a new host cell. The frequent proximity between the C-gene terminators and R-gene initiators in C-protein-controlled RM systems (Figure 2) is suggestive of translational coupling [38,39]. Regarding the hairpins, they were noted in earlier studies, and at least one of them could explain termination products seen in some primer extension experiments [9]. In addition to the overlap and hairpins, in another C-controlled RM system (Eco72I), the C protein itself has an apparent translational regulatory role [25]. Accordingly, we also tested the ability of C.PvuII to affect translation of *pvuIICR *mRNA *in trans*.

## Results

### Translational *lacZ *fusions to *pvuIIC *and *pvuIIR*

**Figure 3:**
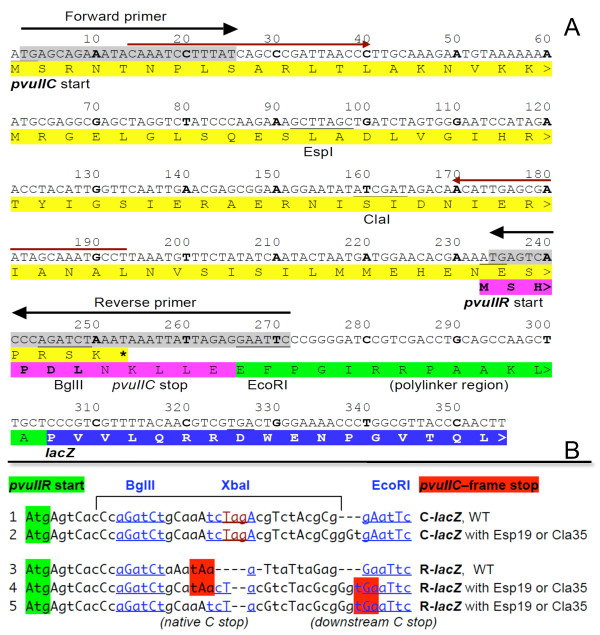
***pvuII-lacZ *transcriptional fusions**. **A**. Sequence from the *pvuIIC *initiator codon to the *pvuIIR*-fused *lacZ *gene. The vector, and source of the polylinker (green) and *lacZ *gene (blue), is pLex3B (ATCC #87200) [58]. The primers indicated by black arrows and gray shading were used to PCR-amplify *pvuIIC *and part of *pvuIIR*; *pvuIIR *retains its native RBS. The *pvuIIC *gene includes two unique sites, ClaI and EspI (equivalent to BlpI), at which different null mutations in *pvuIIC *were generated as previously described [17]. The WT and three different mutants were cloned between the vector XmnI and EcoRI sites such that 'A' of the XmnI site blunt ligated to the 'TG' of the insert on the 5' end to regenerate the *pvuIIC *initiation codon (under the control of the vector's promoter and RBS); the 3' end ligation used the EcoRI site. In derivatives, synthetic oligonucleotides were inserted between the BglII and EcoRI sites (underlined) to fuse *pvuIIC *to *lacZ *or to introduce other changes. The pair of red arrows indicates primers used for mRNA quantitation. **B**. Oligonucleotides used to alter the *pvuIIC-pvuIIR *overlap region. A 30 bp oligonucleotide was cloned between the BglII and EcoRI sites (in the sequence shown in Figure 3) to put *pvuIIC *in-frame with the *lacZ *gene. In each case, the *pvuIIR *initiation codon is highlighted in green, and the *pvuIIC*-frame terminator in red. A unique XbaI site was included to help identify the desired clones and to facilitate shifting the fusion reading frame. A *pvuIIC *stop codon was introduced in some cases (bottom sequences), to restore it to its native location relative to the *pvuIIR *initiator. In lines 1 and 2, the *pvuIIC*-frame terminator is off to the right (not shown).

To explore possible translational coupling, we made *lacZ *translational fusions to the *pvuIIC *and *pvuIIR *genes (Figure 3A; Methods). Importantly, in these fusions, transcription is from P*lacUV5 *and is independent of C.PvuII. Furthermore, the vector's RBS is used, ensuring consistent and efficient translation initiation of *pvuIIC*.

**Figure 4:**
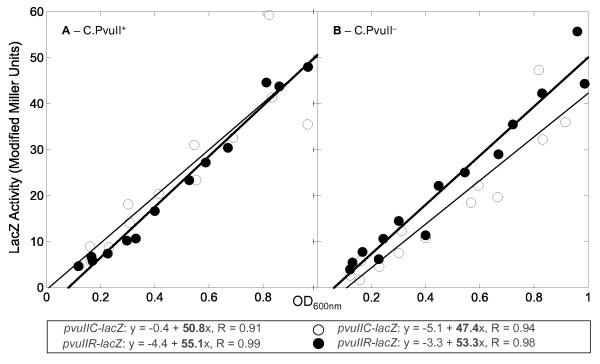
**Expression of *pvuIIC-lacZ *and *pvuIIR-lacZ *translational fusions in the presence and absence of C.PvuII**. Cultures of *E. coli *TOP10 were grown in exponential phase in defined rich medium containing IPTG, and samples were taken at several times for β-galactosidase assays. If the cells are in balanced growth, the plot of activity *vs*. culture density should be linear. The translational fusions were to *pvuIIC *(open circles) or *pvuIIR *(closed circles), and in both cases are transcribed from a C-independent vector promoter. The equations resulting from linear regression are shown. **A**. A compatible plasmid is providing active C.PvuII *in trans*. **B**. As in (A), except the plasmid is providing an inactive version of C.PvuII.

We first measured β-galactosidase activity from WT fusions (see lines 1 and 3 of Figure 3B for junction sequences). Over the course of exponential-phase growth, the plot of activity *vs*. culture density should be linear if the culture is in balanced growth and the assay is in the linear range. The resulting slopes give a precise measure of relative activity. Using this approach, the level of expression of the WT *pvuIIR-lacZ *translational fusion was not significantly different from that of the WT *pvuIIC-lacZ *fusion (Figure 4). Providing WT C.PvuII (or a mutant version) *in trans *from a second, compatible plasmid had little if any effect on the fusions (Figure 4A versus 4B, open versus closed circles). The four slopes are very similar, giving a combined average ± SE of 51.7 ± 1.7 (*i.e*., 3.3% SE). This is inconsistent with *trans*-acting translational effects of C.PvuII protein, unlike the case of C.Eco72I [25].

### Assessing translational coupling of *pvuIIC *and *pvuIIR*

**Figure 5:**
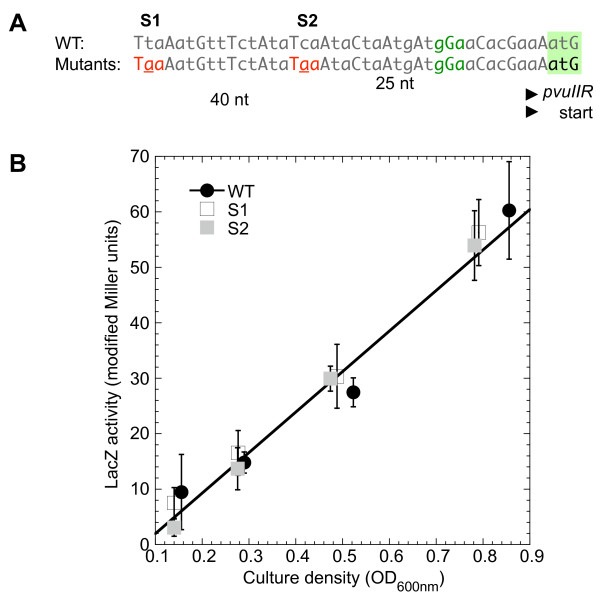
**Stop codons created in *pvuIIC *upstream of *pvuIIR-lac*Z fusion initiation site**. The native *pvuIIC *translation terminator is downstream of the *pvuIIR *initiation codon (see top line of Figure 2). **A**. To test for translational coupling, site-directed mutation was used to introduce *pvuIIC *terminators farther upstream. The *pvuIIR *RBS and intiation codon are indicated in green. The two introduced stop codons were in two independent clones. **B**. *lacZ *activity of the WT (filled circles) and the mutants (open squares have terminator indicated "S1" in part A; filled squares represent S2). The linear fit is to the WT data.

Because coupling is sensitive to the proximity of the termination and start codons of the coupled genes [40], LacZ specific activity was measured in *pvuIIR-lacZ *fusions with the *pvuIIC *stop codon at its native site (+20 nt relative to ATG of *pvuIIR*; Figure 2A) or moved ~20 nt downstream; no effect of this move was seen on translation of *pvuIIR-lacZ *fusions (not shown). We also generated stop codons 25 or 40 bp upstream of the *pvuIIR *initiation codon (Figure 5A), in an otherwise-WT *pvuIIR-lacZ *translational fusion, and measured the LacZ activity (Figure 5B). These stop codons did not detectably affect translation in the *pvuII*R reading frame.

Nevertheless, previous findings [41] and the results from the WT translational fusions indicate that the translation of *pvuIIR *is very similar to that of *pvuIIC *under several conditions, consistent with their translation being coupled. Accordingly we further tested this possibility.

### Frameshift mutation in *pvuIIC *reduces translation of *pvuIIR*

**Figure 6:**
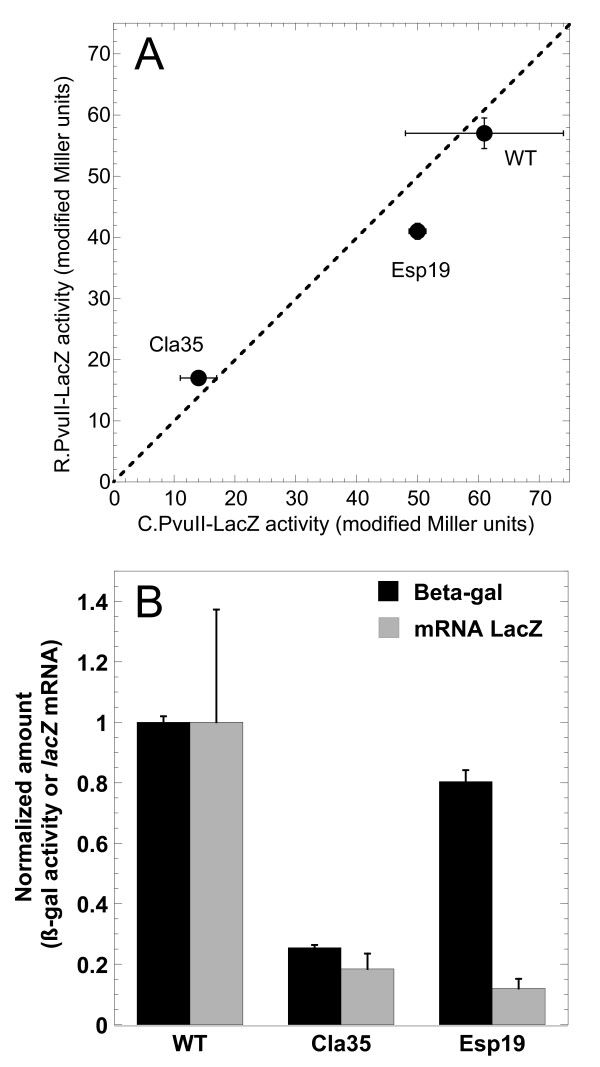
**Effects of *pvuIIC *mutation**. **A**. Comparison of *pvuIIC *and *pvuIIR *translation with WT and mutant variants of *pvuIIC*. The slopes from triplicate experiments such as that shown in Figure 4 are plotted, showing standard errors (where bars are not visible, errors were smaller than the symbol). In this correlogram, activity from the *pvuIIC-lacZ *translational fusion is shown on the x-axis, and that from the *pvuIIR-lacZ *fusion of the same mutant is on the y-axis; if the two fusions for a given variant have equal translation, the point would fall on the dotted line. **B**. Effects of mutation on mRNA levels and translation activity of *pvuIIR-lacZ *fusions. Reporter *pvuIIR *fusions with WT *pvuIIC *upstream, or with the Cla35 (frameshift) or Esp19 (in-frame) *pvuIIC *mutations, were grown in triplicate. Real-time RT-PCR was carried out as described in Methods, using the SYBR green method [60] and primers specific to *lacZ*. Quantitation was based on a standard curve with normalization to *recA *mRNA. Amounts of mRNA (gray bars) are normalized to the level in the strain carrying WT *pvuIIC*. The black bars indicate *lacZ *activity measurements in the same cultures, measured as shown in Figure 4 and normalized to the WT value. Standard errors are shown.

We next determined the effects of mutations within *pvuIIC*, using our system in which transcription is driven from a promoter unaffected by C.PvuII. WT C.PvuII was supplied from a compatible plasmid *in trans*. Figure 6A is a correlogram, showing β-galactosidase expression, with the upstream ORF being WT or mutant *pvuIIC*, and *lacZ *is fused in the *pvuIIC *reading frame (*x-*axis) or in the *pvuIIR *reading frame (*y*-axis). The *pvuIIC *termination codon was, in all cases, in the native location. When *pvuIIC *was WT (up to the point of the fusion; see Figure 3B), translation was roughly equivalent in both reading frames.

Esp19 is an in-frame insertion of one leucine codon into *pvuIIC*, within the first helix of the DNA-binding helix-turn-helix motif [41] (see Figure 3A, and Additional file 1, Figure S1). Like the "WT" fusions, the *pvuIIC*-Esp19 fusions gave similar translational activity in both reading frames. Furthermore, the levels of translation were fairly similar to those when *pvuIIC *was WT.

Cla35 is a frameshift mutation in *pvuIIC *that results in a termination codon ~70 nt upstream of the normal stop codon [17] (see Figure 3A, and Additional file 1, Figure S1). The new stop codon is UAG followed by A, which in one *in vivo *assay system gives a termination rate of about 50% [42]. This mutation led to a roughly fivefold decrease in β-galactosidase expression, relative to the WT fusions, when *lacZ *was fused in either reading frame. This effect appears to be due to transcriptional polarity (see below), but the important point here is that *pvuIIR- *and *pvuIIC-*frame translation changed in parallel.

### Quantitative real-time RT-PCR (QRT-PCR) reveals transcriptional polarity

The parallel fivefold drop in *pvuIIC *and *pvuIIR *translation in the *pvuIIC-*Cla35 frameshift mutant (Figure 6A) could reflect premature translation termination leading to premature transcript termination [43,44]. To test this, relative *lacZ *mRNA levels were determined by QRT-PCR for the WT and both mutant *pvuIIR-lacZ *fusion strains (see lines 3-5 in Figure 3B). In the case of Cla35, the amount of *lacZ *mRNA dropped by the same fivefold amount as did translation (Figure 6B), consistent with transcriptional polarity (and, for that matter, with coupling between the rates of *pvuIIC *and *pvuIIR *translation).

However, surprisingly, the levels of *lacZ *mRNA dropped by about the same extent in the strains bearing an in-frame single-codon insertion (Esp19; Figure 6B). The junction sequences of the Cla35 and Esp19 fusions are identical (line 5, Figure 3B). Esp19 only minimally changed the level of *pvuIIR-lacZ *translation (Figure 6), but this implies that it substantially increased the apparent translational initiation at the downstream *pvuIIR *initiator, relative to WT, as there is only about an eighth as much mRNA (gray bars in Figure 6B).

### Putative hairpins upstream of *pvuIIR *have no obvious effect on its translation

**Figure 7:**
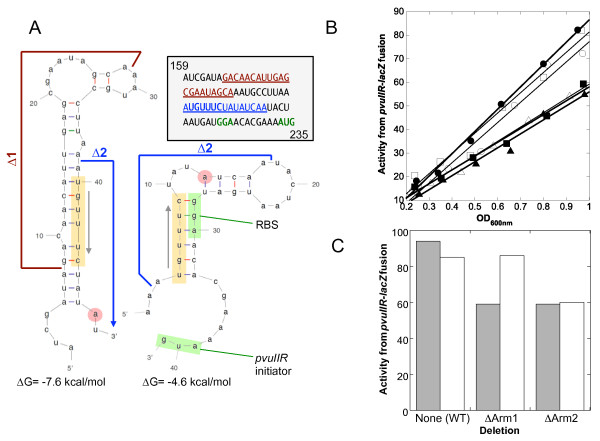
**RNA secondary structure upstream of *pvuIIR***. **A**. Putative alternative hairpins in *pvuIICR *mRNA. The sequence from the *pvuIIC *gene just upstream of *pvuIIR *is shown, with numbering corresponding to that in Figure 3A. As previously described [9], the program MFOLD [61] predicts alternative hairpin structures, the downstream one of which would occlude the RBS (structure on right, green highlight). The orange highlighting shows a sequence shared between the two structures, making them mutually exclusive. The red circle indicates the position of a termination product in previous primer extension reactions [7]. The boundaries of two in-frame deletions are indicated. **B**. Effect of in-frame hairpin arm deletions on translation of *pvuIIR*. The deletions shown in (A) were introduced into the *pvuIIR-lacZ *translational fusion, and β-galactosidase activity was measured as described for Figure 4. This was repeated in the presence of WT (closed symbols, gray bars) or null mutant forms of C.PvuII (open symbols, white bars) provided *in trans *from compatible plasmids. Plot of activity *vs*. culture density, for WT (circles), arm 1 deletion mutant (squares), or arm 2 deletion mutant (triangles). **C**. Slopes from (B) are shown to facilitate comparison.

The *pvuIIR *gene is preceded by two predicted, alternative hairpins ([9]; Figure 7A). Previous primer extension studies [9] were consistent with the presence of the upstream hairpin (left in Figure 7A), as a termination product was seen immediately adjacent to the predicted stem (red circles). These hairpins might regulate translation of *pvuIIR*, because the downstream hairpin (right side of Figure 7A) would occlude the RBS. Occlusion of an RBS by mRNA secondary structure is a major determinant of translational initiation rates [45-49].

If this model is correct, altering Arm 2 (orange-shaded sequence in Figure 7A) should disrupt both hairpins and thus increase *pvuIIR *translation, while altering Arm 1 should disrupt the 5' hairpin, promote formation of the 3' hairpin, and thus decrease translation of *pvuIIR*. We made an in-frame deletion of Arm 2 from the WT construct ("Δ2" in Figure 7A, and Additional file 1, Figure S1) and tested the effects on a *pvuIIR-lacZ *translational fusion (Figure 7B). Rather than the predicted increase in translation, this deletion reduced expression somewhat. [Supplying WT C.PvuII *in trans *had no effect (as expected, since a C-independent promoter was being used).] In contrast, the ΔArm1 deletion (Figure 7A, and Additional file 1, Figure S1) was predicted to reduce *pvuIIR-lacZ *translation, but did so only mildly, and even then only in the presence of WT C.PvuII supplied *in trans *(Figure 7C).

## Discussion

In bacteria such as *E coli*, genes are often transcribed into polycistronic mRNAs [50]. Ribosomes from an upstream gene can reinitiate translation at the next initiator in a process called translational coupling. The extent of dependence on reinitiation (as opposed to initiation by newly-bound ribosomes) varies, but tends to vary inversely with the distance between the termination and reinitiation codon [51]. In most polycistronic operons, termination codons are close to the initiation codon of the downstream gene; in many cases they even overlap [37], as in the BcnI and AhdI R-M systems (Figure 2A). A high degree of coupling results in coordinated expression of the genes, as illustrated by the *gal *operon [43,52].

In RM systems that rely on upstream C genes to mediate the delay in REase expression, allowing the MTase time to protect a new host's chromosome [28]; translational coupling could play an important role in the regulatory design. The C and R genes of another C-activated RM system, Esp1396I, are in fact translationally coupled [44]. Furthermore, as shown in Figure 1, translation of *pvuIIC *from the early *pvuIICR *transcripts relies on a poor RBS (a feature not shared by most of the C genes). Translational coupling of *pvuIIR *to *pvuIIC *would ensure that very little R.PvuII is made from the early transcripts (though in rare cases transcripts lacking RBSs in the leader can still be translated efficiently [53]). Once C.PvuII begins to accumulate, it not only generates a positive feedback loop by activating a second promoter upstream of *pvuIIC*, but translation of these later transcripts should be much more efficient as they are leaderless [7] and do not rely on the weak RBS.

However our evidence provides no consistent evidence that the translation of *pvuIIR *is coupled to that of *pvuIIC*. Most significantly, moving the *pvuIIC *termination codon in either direction relative to the *pvuIIR *initiator (Figures 3B, 5) had no apparent effect. In contrast, coupled genes are quite sensitive to the stop-start codon spacing (*e.g*., see Additional file 1, Figure S2, replotted from data in [40]).

This lack of coupling is consistent with previous work from our laboratory. We had demonstrated a sharp decrease in the expression of *pvuIIR *in each of four *pvuIIC *null mutants [17], including two in-frame (EspI) and two frameshift (ClaI) mutants (two of which were used in the current study). These mutants showed greatly-impaired restriction of infection by unmethylated bacteriophage λ, or of transformation by plasmid DNA, and the *in vitro *R.PvuII activity was ~10^4^-fold less than in strains that carried the intact parental plasmid. Providing *pvuIIC in trans *had no effect on the WT strain, but providing it to any of the *pvuIIC *mutants resulted in full restoration of *in vivo *or *in vitro *R.PvuII activity. However boosting transcription of frameshift mutants, that terminate translation ~70 nt upstream of the *pvuIIR *initiation codon (Figure 3A, and Additional file 1, Figure S1), would not have restored a flow of translating ribosomes to the *pvuIIR *initiator, so these results conflict with coupling.

C proteins themselves can have regulatory effects at the translational level. In the Eco72I RM system [25], providing C.Eco72I *in trans *to *eco72IR-lacZ *translational fusions restored REase gene expression even when the upstream *eco72IC *gene included a frameshift mutation. However, in the case of PvuII, neither *pvuIIC-lacZ *nor *pvuIIR-lacZ *translational fusions showed substantial effects of supplying C.PvuII *in trans*.

Hairpins in the RNA that occlude RBSs can reduce translation initiation [45,47-49], though initiating ribosomes have the capacity to unfold mRNA [54] and may also be prepositioned to slide into place when an RNA hairpin spontaneously unfolds [46]. In-frame deletions in *pvuIIC*, that would affect predicted alternative hairpins upstream of *pvuIIR *and thus alter the availability of the *pvuIIR *RBS (Figure 7A), had no major effect (Figure 7B, C). However, the hairpins might nonetheless reduce the entry of new ribosomes at the *pvuIIR *RBS, while not affecting progress of translating ribosomes. Searching for potential RBS-occluding hairpins in other C-dependent R-M systems showed that 9/11 had such hairpins with ΔG values ≤ -4.0 kcal/mol (Additional file 1, Figure S3). For comparison, the equivalent PvuII hairpin has a predicted ΔG of -4.6 kcal/mol.

## Conclusion

The genes for RM systems are often associated with mobile genetic elements, and temporal control is critical following the entry of these genes into a new cell, to avoid restriction of the host's chromosome. The role of translation-level regulation in this process has not been well studied. In examining the PvuII RM system, we found that translation of the downstream REase gene (*pvuIIR*) is independent of that of the upstream gene for the autogenous activator/repressor (*pvuIIC*), at least under the conditions used. This independence was despite the overlapping genes and the presence of putative RNA hairpins involving the *pvuIIR *RBS. This suggests that the temporal control of PvuII transcription [28], together with the repair capabilities of the bacteria [55-57], is sufficiently robust to protect the new host cell following PvuII gene transfer.

## Methods

### Cloning and generation of mutants

Table 1 and Additional file 1, Table S1 show, respectively, the plasmids and oligonucleotides that were used in this study. The *Cla *and *Esp *mutants of *pvuIIC *were generated previously by this lab [17]. p*PvuRM*3.4 contains the WT PvuII R-M system as a 3.4 kb fragment in vector pBR322 [12], and this R-M system includes two unique enzyme sites in the *pvuIIC *ORF, ClaI and EspI (currently available as the isoschizomer BlpI) (see Figure 3A, and Additional file 1, Figure S1). At the ClaI site, filling in the 5' extensions with Klenow polymerase or digestion using mung bean nuclease, followed by religation, generates frameshift mutations. EspI yields a 3-bp overhang (GC/TNAGC), so fill-in or digestion creates an in-frame mutation [17].

**Table 1:** Plasmids used in this study

Name	Relevant Feature(s)	Reference
pDK200	WT *pvuIIC *gene with its own promoter, Δ*pvuIIM*,	[7]
pDK201	Δ*pvuIIR; *Tet^R^, pACYC177	[7]
pDK201-Esp33	pDK200 with *pvuIIC-*Esp19 in-frame insertion	This study
pDK201-Cla35	pDK200 with *pvuIIC-*Esp33 in-frame insertion	This study
pPvuIIRM3.4	pDK200 with *pvuIIC-*Cla35 frameshift mutation	[12]
pPvuIIRM3.4- Esp19	WT PvuII R-M system; Amp^R^, pBR322	[17]
pLex3B	pPvuIIRM3.4 with *pvuIIC-*Esp19 in-frame insertion	[58]
	Plasmid from ATCC (#87200) used to create translational fusions, to *lacZ*; Amp^R^, pMB1 origin	

For this study, DNAs from WT *pvuIIC*, mutant Esp19 (with insertion of a leucine codon) and mutant Cla35 (frameshift insertion), were purified using the QIAprep plasmid kit (Qiagen, Germantown, MD). Oligonucleotide primers were purchased from Integrated DNA technology company (IDT, Coralville, IA). The amplified region began upstream of the *pvuIIC ***ATG **initiator and ended a few codons into *pvuIIR *(including the entire overlap between *pvuIIC *and *pvuIIR*, ~285 bp). High fidelity Pfx polymerase (New England Biolabs, Ipswich, MA) was used to amplify the fragment, which was then purified using a Qiagen gel extraction kit.

The amplified fragments were cloned into **TOPO 2.1 **vector (Invitrogen, Carlsbad, CA), and ligation products were used to transform **TOP-10 **chemically competent *E. coli *(Invitrogen). Transformants were plated onto LB agar with 100 μg/ml ampicillin. Colonies were inoculated into LB broth with appropriate antibiotics, and clones demonstrating the expected restriction pattern were confirmed by sequencing.

### LacZ translational fusions

Inserts from sequence confirmed clones in TOPO 2.1 were again amplified using a second set of primers (black arrows in Figure 3A). The secondary amplification began with '**TG' **of the **ATG **initiator of *pvuIIC *at the 5' end, and extended into *pvuIIR *(downstream of the *pvuIIC *termination codon). An EcoRI site was added to the 3' end of the insert. Plasmid pPvuIIRM3.4 carries the WT PvuII RM system in vector pBR322 [12], and *pvuIIC *contains unique restriction sites (ClaI and EspI/BlpI) that were used to generate mutants [17] (Figure 3A). Wild type and mutant versions of pPvuIIRM3.4 were used as templates. The amplified segments were cloned in-frame with *lacZ *in vector pLEX3B [58]. PCR amplification used high fidelity Pfx polymerase (New England Biolabs). The EcoRI digested fragment was purified by electrophoresis and the Qiagen gel extraction kit. A similar protocol was followed for all three mutants and the wild type.

We prepared fusions in which the *pvuIIC *initiation codon was the optimal distance from the RBS of **pLEX3B **(ATCC # 87200, ATCC, Manassas, VA). Insert DNA was cloned between the XmnI and EcoRI site of the **pLEX3B **vector, such that on the 5' end 'A' of the XmnI site was blunt ligated to the 'TG' of the insert; the 3' end ligation used the EcoRI site. To facilitate converting these clones from *pvuIIC *to *pvuIIR *being in-frame with *lacZ*, a 30 bp oligonucleotide was acquired with a BglII site at the 5' end (sense strand) and EcoRI at the 3' end (see Figure 3B). In the middle was a unique XbaI site to facilitate identification of the desired clones. This 30 mer duplex was cloned between the BglII and EcoRI sites of the *pvuIIR*-*lacZ *fusions (see Figure 3B).

The WT *pvuIIC *translational fusion contains the full ORF and few codons from *pvuIIR *in-frame with *lacZ*. We removed the stop codon from the *pvuIIC *ORF. In the Cla35 frameshift mutant of *pvuIIC*, *lacZ *is in-frame with the shifted *pvuIIC *reading frame (though there are intervening nonsense codons). Two nucleotides (AG) were deleted to shift the frame, such that *pvuIIR *was in-frame with *lacZ*. This shifted the stop codon for *pvuIIC *by few codons relative to the *pvuIIR *initiator, so we used site directed mutagenesis to restore the stop codon to its original position by substitution. Primers (IDT) were designed for this purpose, to function with the Stratagene QuickII site-directed mutagenesis protocol and kit (Agilent, Santa Clara, CA).

Ligation products were used for transformation as described above, except that IPTG (1 mM), and X-gal (bromo-chloro-indolyl-galactopyranoside, 40 μg/ml) were also added to the agar. Transformants that turned blue were patched and also inoculated into LB broth with the appropriate antibiotics. Putative *lacZ *translational fusions were isolated and confirmed by restriction-digestion as well as colony PCR. Clones that demonstrated the desired restriction pattern were confirmed by sequencing.

### Hairpin mutants

We prepared in-frame arm1 and arm 2 deletions in the predicted hairpins (Figure 7A) using site-directed mutagenesis kit and protocols as described above. Again this set was confirmed by sequencing (MWG, High Point, NC).

### Beta-galactosidase assays

We measured hydrolysis of the substrate *ο*-nitrophenyl-β-D-galactoside (ONPG). Overnight cultures were grown at 37°C in MOPS rich defined medium (TEKNOVA, Hollister, CA), in the presence of tetracycline (10 μg/ml) and the inducer IPTG (1 mM). This culture was diluted 1:100 into the same medium, without tetracycline, and grown to exponential phase. Samples were collected at regular intervals and the optical density (OD) at 600 nm was measured for every 1 ml sample. Two drops of chloroform and one drop of 0.1% SDS were added, and the sample vortexed for 10 s to open the cells. Samples were placed in a 28°C water bath for 5 min, and 200 μl ONPG (4 mg/ml) were added. Reactions were stopped with 500 μl 1 M Na_2_CO_3_, and OD_420 nm _was determined. The formula of Miller [59] was used to calculate units of β-galactosidase. Miller units were plotted against the culture OD_600 nm_. The slopes from linear regression indicate relative specific activity; linearity indicates that the culture was in balanced growth.

### RNA isolation and quantitation

Cultures were grown as described above, to an OD _600 nm _of ~0.3, in the presence of IPTG (1 mM). Samples were taken from the flasks and directly added to two volumes of RNA stabilization buffer (RNA Protect Bacteria Reagent, Qiagen, Valencia, CA). Samples were mixed and left at room temperature for 10 min. The RNeasy miniprep kit (Qiagen) was used to isolate total RNA. Cells in stabilization buffer were harvested by centrifugation at 4°C for 15 min at 5,000 rpm. Pellets were resuspended in 1× TE buffer containing lysozyme (400 μg/ml) after removing the supernatant. Ethyl alcohol was added to precipitate the RNA, which was then dissolved and loaded onto the columns. Following column washing and elution, RNA was treated with RQ1 RNAse-free DNAse (Promega, Madison, WI) to remove DNA. cDNA was synthesized using total RNA as template, random hexamers (Invitrogen), and ImPromII reverse transcriptase (Promega). The random primers were annealed at 25°C for 5 min, and the first strand was then extended at 42°C for 1 h. The reverse transcriptase was then inactivated by heating at 70°C for 1 h.

Red arrows in Figure 3A indicate the primer pair used to quantitate *pvuIIC *cDNA. Dilutions of cDNA were tested to determine the maximally-efficient concentration for amplification, and also the efficiency of each primer pair. *recA *expression was also measured, to provide an internal baseline. Cycle threshold (Ct) values were determined using a Roche Light Cycler with SYBR Green. Melting curve analysis was used to confirm the formation of specific products. The standard curve method was used to determine the relative amounts of mRNA, which were normalized to *recA *[60].

## Authors' contributions

MK carried out the strain constructions and experimental analysis, participated in the experimental design, and drafted the manuscript. RB conceived of the study, participated in its design and coordination, helped to draft the manuscript, and generated the figures. Both authors read and approved the final manuscript.

## Supplementary Material

Additional file 1 **Supplemental Information. Figure S1. Location of mutations relative to features of C.PvuII**. The amino acid sequence of C.PvuII is shown in single-letter code (GenBank AAA96335.1). The DNA-binding helix-turn-helix motif contains the EspI (BlpI) site; fill-in or resection followed by religation duplicates or removes the leucine in the "SLA" sequence. Orange shading indicates the subunit interface, from comparison to the crystal structure for an orthologous C protein [20]. This interface spans the ClaI site used to generate frameshift mutations. The two in-frame deletions of predicted RNA hairpin elements (see Figure 7A) are indicated as Δ1 and Δ2. The position of the alternate reading frame *pvuIIR *initiation codon is indicated. **Figure S2. Effects of distance between termination and initiation codons on translational reinitiation**. This is a replotting of data from [40]. The *lacZ *gene was introduced into *E. coli *with varied spacing between the *lacZ *initiation codon and the terminator for the upstream ORF. **Figure S3. Putative RNA hairpins upstream of REase gene initiation codon in selected C-dependent R-M systems**. C-dependent R-M systems, from among those shown in Figure 2, were analyzed for potential hairpin structures upstream of the REase gene initiation codon. The 40 nt upstream of each initiator were submitted to MFOLD [61], and the most stable structure returned in each case is shown. The number in parentheses is the predicted ΔG of folding, in kcal/mol. The Esp1396I structure may be further stabilized by its GNRA loop [62].The initiator codon and predicted RBS are highlighted in green. Secondary structures are shown for comparison, whether or not they are likely to be present a significant fraction of the time. Click here for file
