# Supplementary material for: Translational independence between overlapping genes for a restriction endonuclease and its transcriptional regulator
Source: BMC Mol Biol. 2010 Nov 19;11:87. doi: 10.1186/1471-2199-11-87 (PMC2997769; doi:10.1186/1471-2199-11-87)
Supplement: Additional file 1 — Supplemental Information. Figure S1. Location of mutations relative to features of C.PvuII. The amino acid sequence of C.PvuII is shown in single-letter code (GenBank AAA96335.1). The DNA-binding helix-turn-helix motif contains the EspI (BlpI) site; fill-in or resection followed by religation duplicates or removes the leucine in the "SLA" sequence. Orange shading indicates the subunit interface, from comparison to the crystal structure for an orthologous C protein [20]. This interface spans the ClaI site used to generate frameshift mutations. The two in-frame deletions of predicted RNA hairpin elements (see Figure 7A) are indicated as Δ1 and Δ2. The position of the alternate reading frame pvuIIR initiation codon is indicated. Figure S2. Effects of distance between termination and initiation codons on translational reinitiation. This is a replotting of data from [40]. The lacZ gene was introduced into E. coli with varied spacing between the lacZ initiation codon and the terminator for the upstream ORF. Figure S3. Putative RNA hairpins upstream of REase gene initiation codon in selected C-dependent R-M systems. C-dependent R-M systems, from among those shown in Figure 2, were analyzed for potential hairpin structures upstream of the REase gene initiation codon. The 40 nt upstream of each initiator were submitted to MFOLD [61], and the most stable structure returned in each case is shown. The number in parentheses is the predicted ΔG of folding, in kcal/mol. The Esp1396I structure may be further stabilized by its GNRA loop [62].The initiator codon and predicted RBS are highlighted in green. Secondary structures are shown for comparison, whether or not they are likely to be present a significant fraction of the time. [file 1471-2199-11-87-S1.PDF]

Supplemental information for:

Translational Independence Between Overlapping Genes for a Restriction Endonuclease and its Transcriptional Regulator

Meenakshi K. Kaw[1] and Robert M. Blumenthal[1,2,*]

[1]Department of Medical Microbiology and Immunology, University of Toledo Health Science Campus, 3100 Transverse Drive, Toledo, OH 43614-2598, USA
[2]Program in Bioinformatics and Proteomics/Genomics, University of Toledo Health Science Campus, 3100 Transverse Drive, Toledo, OH 43614-2598, USA

*Corresponding author

E-mail: Robert.Blumenthal@utoledo.edu
Meenakshi.Kaw@utoledo.edu

BMC Molecular Biology

Table S1. Oligonucleotides used in this study

Oligo-nucleotide	Sequences (all 5’→3’)	Use
PvuLexFor	TGAGCAGAAATACAAATCCTTTATCAGC	Cloning PvuII genes into pLex3B
PvuLexRev	CGGGATCCTCTAATAATTTATTTAGATCTGGGTGACTCATT	
PvuTAAFor	CCCAGATCTGCATAACTACGTCTACGCG	Restoring *pvuIIC* TAA to original location
PvuTAARev	CGCGTAGACGTAGTTATGCAGATCTGGG	
*pvuIIC*For	CAAATCCTTTATCAGCCCGATTAACCC	QRT-PCR of *pvuIIC* mRNA
*pvuIIC*Rev	AGGCATTTGCTATTCGCTCAATGT	
*recA*For	TAACCTGAAGCAGTCCAACACGCT	QRT-PCR of *recA* mRNA
*recA*Rev	TTGTTCTTCACCACTTTCACGCGG	
S1TAAFor	CGAATAGCAAATGCCTAAATGTTTCTATATCAATAC	Creating stop codons in *pvuIIR-LacZ* fusions upstream of R-initiation site.
S1TAARev	GTATTGATATAGAAACATTTAGGCATTTGCTATTCG	
S2TAAFor	GCCTTAAATGTTTCTATATAAATACTAATGATGGAACAC	
S2TAARev	GTGTTCCATCATTAGTATTTATATAGAAACATTTAAGGC	
S3TAAFor	CTATACAATACTAATGATGTAACACGAAAATGAGTCACCCAG	
S3TAARev	CTGGGTGACTCATTTTCGTGTTACATCATTAGTATTGTATAG	

Figure S1. Location of mutations relative to features of C.PvuII. The amino acid sequence of C.PvuII is shown in single-letter code (GenBank AAA96335.1). The DNA-binding helix-turn-helix motif contains the EspI (BlpI) site; fill-in or resection followed by religation duplicates or removes the leucine in the “SLA” sequence. Orange shading indicates the subunit interface, from comparison to the crystal structure for an orthologous C protein [1]. This interface spans the ClaI site used to generate frameshift mutations. The two in-frame deletions of predicted RNA hairpin elements (see Fig. 7A) are indicated as Δ1 and Δ2. The position of the alternate reading frame *pvuIIR* initiation codon is indicated.

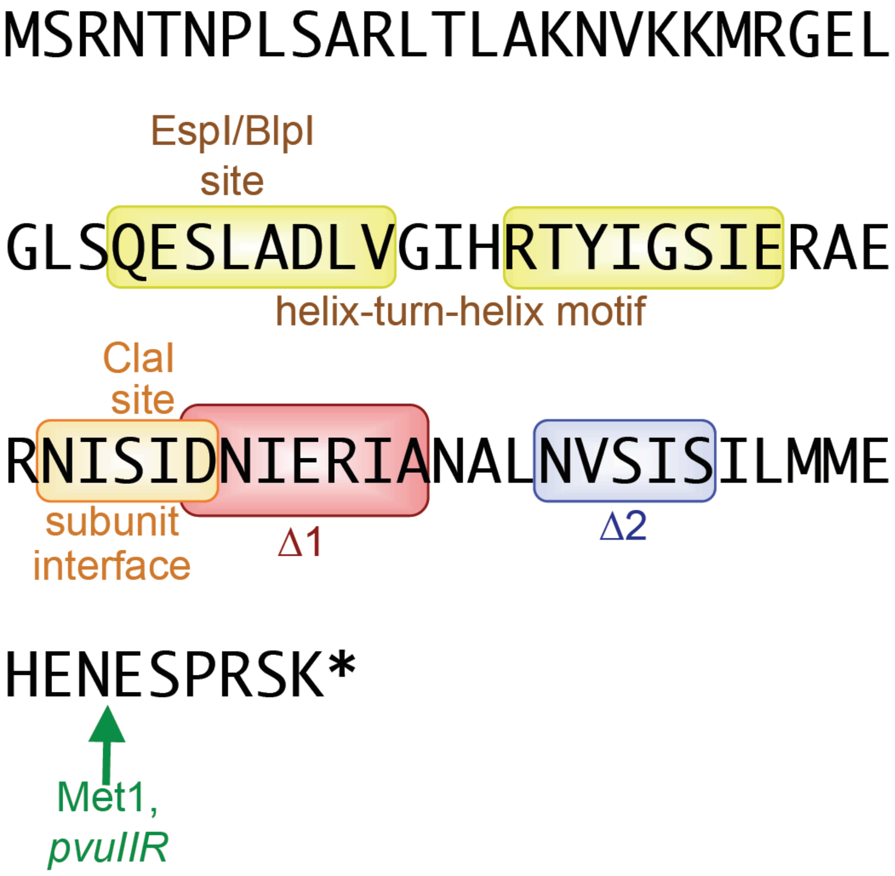

Figure S2. Effects of distance between termination and initiation codons on translational reinitiation. This is a replotting of data from [2]. The *lacZ* gene was introduced into *E. coli* with varied spacing between the *lacZ* initiation codon and the terminator for the upstream ORF.

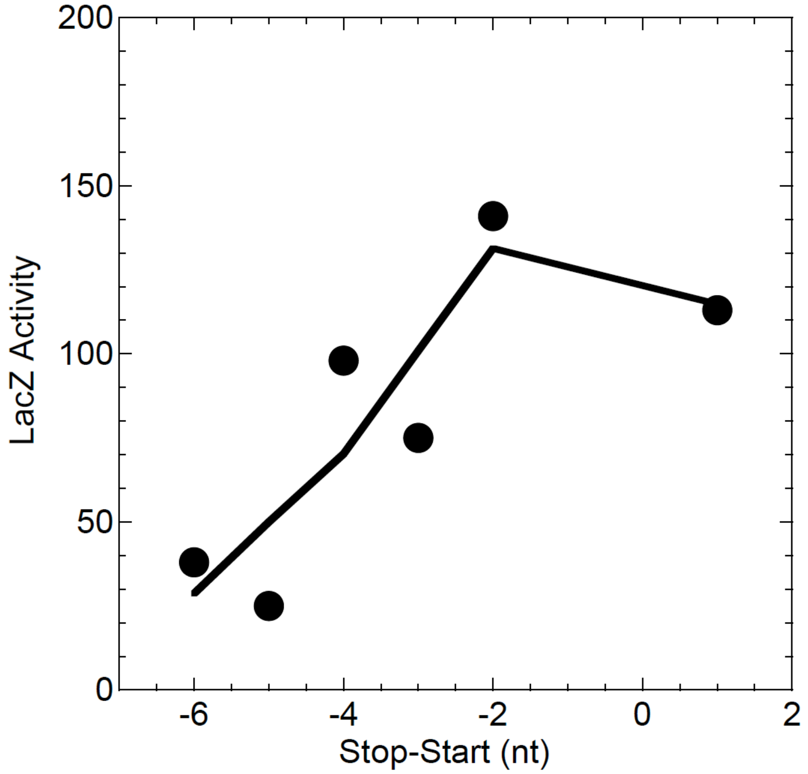

Figure S3. Putative RNA hairpins upstream of REase gene initiation codon in selected C-dependent R-M systems. C-dependent R-M systems, from among those shown in Fig. 2, were analyzed for potential hairpin structures upstream of the REase gene initiation codon. The 40 nt upstream of each initiator were submitted to MFOLD [3], and the most stable structure returned in each case is shown. The number in parentheses is the predicted ∆G of folding, in kcal/mol. The Esp1396I structure may be further stabilized by its GNRA loop [4].The initiator codon and predicted RBS are highlighted in green. Secondary structures are shown for comparison, whether or not they are likely to be present a significant fraction of the time.

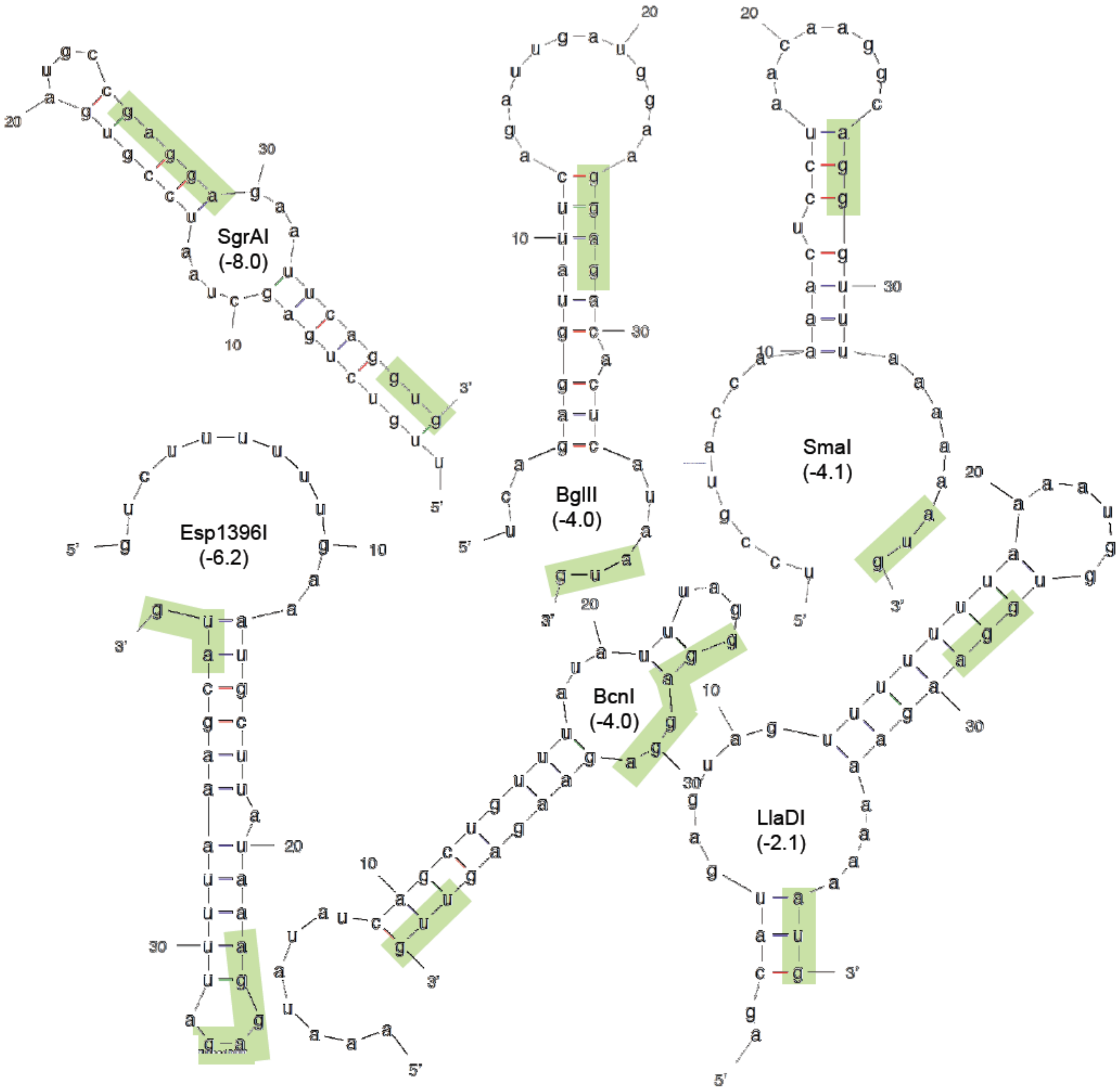

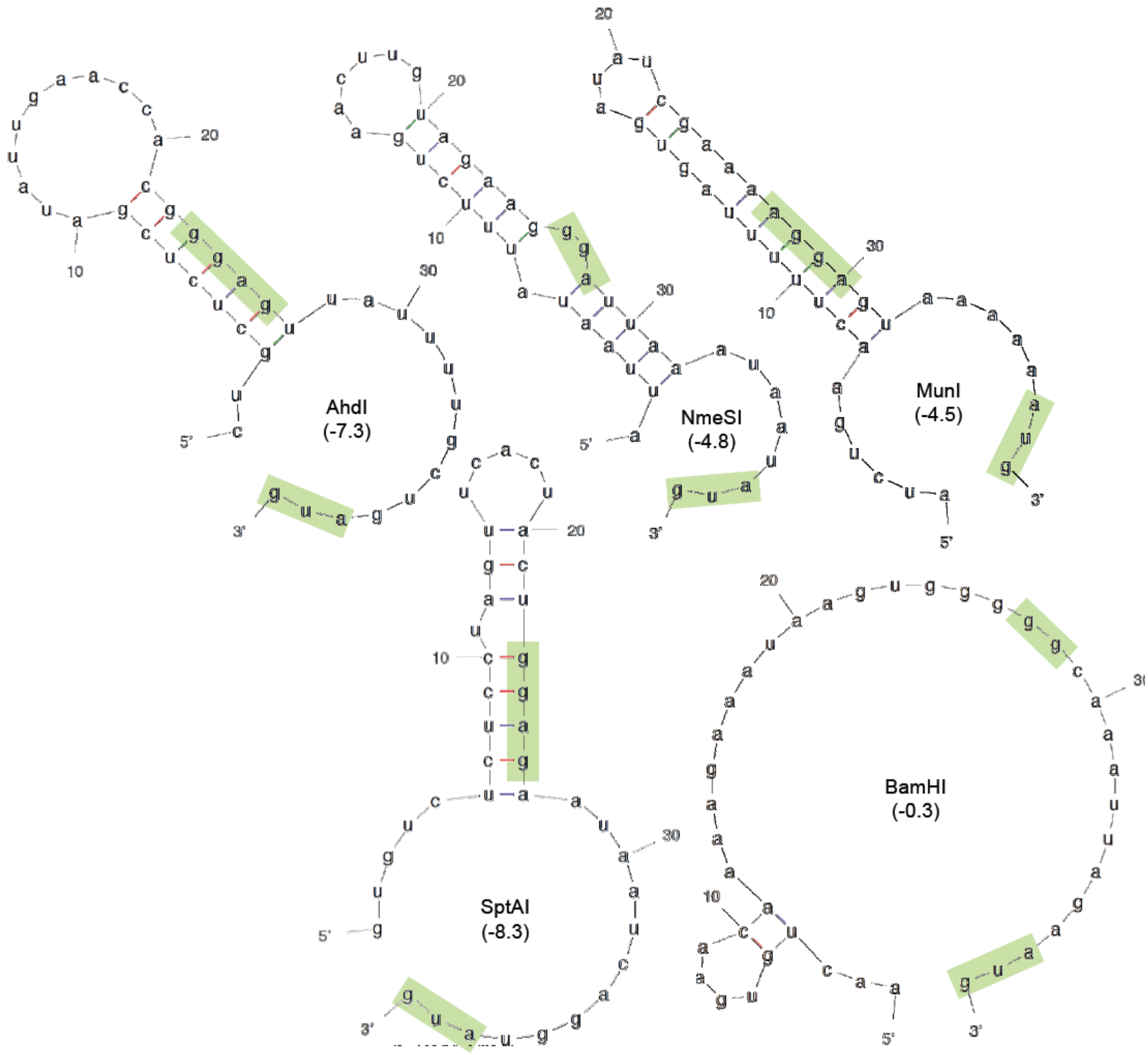

References

1. McGeehan JE, Streeter SD, Papapanagiotou I, Fox GC, Kneale GG: **High-resolution crystal structure of the restriction-modification controller protein C.AhdI from Aeromonas hydrophila**. *J Mol Biol* 2005, **346**(3):689-701.
2. van de Guchte M, Kok J, Venema G: **Distance-dependent translational coupling and interference in Lactococcus lactis**. *Mol Gen Genet* 1991, **227**(1):65-71.
3. Zuker M: **Mfold web server for nucleic acid folding and hybridization prediction**. *Nucleic Acids Res* 2003, **31**(13):3406-3415.
4. Heus HA, Pardi A: **Structural features that give rise to the unusual stability of RNA hairpins containing GNRA loops**. *Science* 1991, **253**(5016):191-194.
